# Baseline Difference in Quantitative Electroencephalography Variables Between Responders and Non-Responders to Low-Frequency Repetitive Transcranial Magnetic Stimulation in Depression

**DOI:** 10.3389/fpsyt.2020.00083

**Published:** 2020-02-27

**Authors:** Premysl Vlcek, Martin Bares, Tomas Novak, Martin Brunovsky

**Affiliations:** ^1^ National Institute of Mental Health, Klecany, Czechia; ^2^ Third Faculty of Medicine, Charles University, Prague, Czechia

**Keywords:** major depressive disorder, repetitive transcranial magnetic stimulation, quantitative electroencephalography, LORETA, EEG asymmetry

## Abstract

Repetitive transcranial magnetic stimulation (rTMS) is an effective treatment for depressive disorder, with outcomes approaching 45–55% response and 30–40% remission. Eligible predictors of treatment outcome, however, are still lacking. Few studies have investigated quantitative electroencephalography (QEEG) parameters as predictors of rTMS treatment outcome and none of them have addressed the source localization techniques to predict the response to low-frequency rTMS (LF rTMS). We investigated electrophysiological differences based on scalp EEG data and inverse solution method, exact low-resolution brain electromagnetic tomography (eLORETA), between responders and non-responders to LF rTMS in resting brain activity recorded prior to the treatment. Twenty-five unmedicated depressive patients (mean age of 45.7 years, 20 females) received a 4-week treatment of LF rTMS (1 Hz; 20 sessions per 600 pulses; 100% of the motor threshold) over the right dorsolateral prefrontal cortex. Comparisons between responders (≥50% reduction in Montgomery-Åsberg Depression Rating Scale score) and non-responders were made at baseline for measures of eLORETA current density, spectral absolute power, and inter-hemispheric and intra-hemispheric EEG asymmetry. Responders were found to have lower current source densities in the alpha-2 and beta-1 frequency bands bilaterally (with predominance on the left side) in the inferior, medial, and middle frontal gyrus, precentral gyrus, cingulate gyrus, anterior cingulate, and insula. The most pronounced difference was found in the left middle frontal gyrus for alpha-2 and beta-1 bands (p < 0.05). Using a spectral absolute power analysis, we found a negative correlation between the absolute power in beta and theta frequency bands on the left frontal electrode F7 and the change in depressive symptomatology. None of the selected asymmetries significantly differentiated responders from non-responders in any frequency band. Pre-treatment reduction of alpha-2 and beta-1 sources, but not QEEG asymmetry, was found in patients with major depressive disorder who responded to LF rTMS treatment. Prospective trials with larger groups of subjects are needed to further validate these findings.

## Introduction

Major depressive disorder (MDD) is one of the most common psychiatric disorders resulting in a lifetime disability. Despite recent advances in psychopharmacology, up to 30% of patients do not respond to antidepressant medications (1). Due to this limitation, several other therapeutic potentials have been introduced. Repetitive transcranial magnetic stimulation (rTMS) is a minimally invasive method that delivers magnetic pulses through a coil placed over the scalp. These pulses induce an electric field in the target cortex regions associated with alterations in neurotransmitters’ release and metabolism or gene expression (2, 3). Up to 20–40% of pharmaco-resistant patients with MDD respond well to rTMS (4). rTMS in MDD treatment has mostly been applied by delivering stimulation at a high frequency (5-20 Hz; HF rTMS) to the left dorsolateral prefrontal cortex (DLPFC) for a period of 2 to 9 weeks (5). A similar effect may be achieved with a low-frequency (1 Hz, LF rTMS) stimulation over the right DLPFC (6). In order to improve clinical outcomes for patients, it is important to assess the potential response to rTMS treatment prior to its application.

Antidepressant rTMS therapy usually utilizes the left-sided DLPFC high-frequency stimulation and rarely the right-sided DLPFC low-frequency stimulation. The rationale being that the former mainly has an excitatory net effect while the latter, an inhibitory net effect. The theoretical reasoning behind the effort to enhance the activity or inhibition of one of the key neuronal limbic structure (based on its connectivity profile to a sub-system including the anterior cingulum, hippocampus, or amygdala), is based on several findings that show that the left-sided DLPFC activity is increased after antidepressant therapy (7–10). In terms of inter-hemispheric connectivity, the ascertainment for post-treatment increase in left-sided activity is consistent with findings that confirm the reduced right-sided DLPFC activity after treatment for the depressive disease (11, 12).

In order to improve personalized treatment for MDD, studies have attempted to detect biomarkers derived from quantitative electroencephalography (QEEG)—for in-depth review see (13, 14). The individual findings can be broken down according to the QEEG variables detected and their frequency band. Typical QEEG variables (alpha, beta, and theta bands) with a predictive value include: relative or absolute alpha, beta and theta power, hemispheral alpha asymmetry, frontal or parietal current source density, beta or theta cordance, coherence, and connectivity (14–22). As long as delays in finding effective treatments through trial and error continue to pose a burden on patients, studies that focus not only on discriminating patients from the healthy subjects but also predict treatment outcomes, are of particular importance. A recent study showed that patients responding to treatment with escitalopram exhibited elevated absolute alpha power in their left hemisphere while non-responders demonstrated the opposite (23). Greater right frontal alpha is associated with response to escitalopram and sertraline, but only for females (24, 25) while less alpha current source density in posterior areas has been associated with nonresponse in unmedicated depressed patients (26, 27). Moreover, successful antidepressant treatment of patients with more severe depression is accompanied by an increase in the left DLPFC and amygdala upper alpha EEG activity (28). Initially, larger beta spectral power values of EEG were associated with the high manifestation of residual depressive symptoms and non-responsivity after the treatment (29, 30), and the smaller beta activity at the frontotemporal region was associated with greater reductions in depressive psychopathology after paroxetine treatment (15).

Several recent QEEG studies have partially demonstrated the ability to predict rTMS treatment response (14, 31). Among the findings, a non-response or poorer response to treatment is characterized as: a change of power in theta (31–33), alpha (34), and beta (30) bands in the frontal cortex, prefrontal beta or theta cordance; decrease/unchanged between the baseline and first week of rTMS application (31, 35, 36); slowing of the anterior alpha peak or a decrease in its frequency (31); larger amplitude of evoked potential P300 (31); no increase in the Lempel-Ziv complex (from minute 1 to 2) in the alpha band (37); and a change in the beta phase-locking values (38).

Limited clinical data are available for the QEEG predictors of LF rTMS (1 Hz) treatment in MDD. For example, Valiulis et al. (39) found a marked shift of frontal alpha power asymmetry toward the right hemisphere, while no major effects were detected in the basic EEG band power. In our previous study, we found that responders to LF rTMS had a significantly reduced prefrontal theta cordance value after one week (40). However, measuring the reduction in cordance value relies on the observable change, once the therapy has begun. Therefore, it does not reflect the pre-treatment difference that indicates an antidepressant response to LF rTMS therapy. Due to the lack of studies on the pre-treatment difference between the responders and the non-responders for antidepressant treatment with LF-rTMS, we analyzed the baseline difference of EEG for selected frequency bands in the interhemispheric pairs of electrodes. We also aimed to identify the brain regions that distinguish responders from non-responders at baseline, before the LF rTMS treatment. For this purpose, we used an exact low-resolution brain electromagnetic tomography (eLORETA) in order to acquire the three-dimensional tomography of brain electrical activity (current densities) and localized multiple distributed cortical sources in different frequency bands from the resting scalp-recorded EEG data.

## Methods

### Subjects

For this study, we used data of 25 adult inpatients aged 18–65, that had participated in previously published studies (40, 41) (EudraCT no. 2005–000826-22) and were allocated to the rTMS arm. Patients included were with a positive diagnosis of MDD (recurrent or single episode) without psychotic symptoms according to the DSM IV criteria (42), confirmed using the Czech version 5.0.0 of the Mini-International Neuropsychiatric Interview (43) and had fulfilled at least stage I criteria for resistant depression (≥1 adequate antidepressant treatment in current episode) according to Thase and Rush (44). Only subjects who scored at least 20 points on the Montgomery-Åsberg Depression Rating Scale (MADRS) (45) and four points on the Clinical Global Impression (CGI) (46), were recruited. The exclusion criteria included suicidal risk, severe, and unstable medical illness (cardiovascular disease, neoplasms, endocrinology disorders, etc.), and neurological disorders (epilepsy and head trauma with loss of consciousness). We did not include any subjects who received electroconvulsive therapy within the 3 months prior to the start of the study and any subjects taking clozapine, olanzapine, lithium, carbamazepine, or valproate, which may affect EEG recordings. Evaluation of the adequacy of previous medication in the index episode was based on the Antidepressant Treatment History Form (47) with a score of at least 3 (more than 4 weeks of treatment inadequate dose). Standard physical examination, medical history evaluation, psychiatric examination, biochemistry, and EEG were performed in order to exclude risks and somatic or psychiatric comorbidities.

Prior to the study, the purpose and procedures of the research were carefully explained to the patients after which each participant provided his/her informed consent. The study was approved by the ethical committee of the Prague Psychiatric Centre/National Institute of Mental Health. The design and all procedures adhered to the latest version of the Declaration of Helsinki and ICH/Good Clinical Practice guidelines.

### Treatment and Clinical Assessment

Following an initial washout period (5–9 days of the antidepressant-free period), eligible subjects received a 4-week treatment with low-frequency rTMS (1 Hz) consisting of twenty 10-min sessions (600 pulses per session) with a 100% stimulation intensity of the resting motor threshold. The rTMS was applied to the right DLPFC at a point 5 cm anterior in a parasagittal line to the motor threshold location (the left abductor pollicis brevis muscle) with the coil held tangentially to the scalp and its handle pointing back and away from the midline at 45°. The rTMS was delivered by a Magstim Super Rapid stimulator (Magstim, Whitland, UK) with an air-cooled, figure-eight 70-mm coil.

Anxiolytics and hypnotics, taken before the start of the rTMS treatment, were permitted in stable doses and not allowed eight hours prior to an EEG recording. No antidepressants, antipsychotics or anticonvulsants were permitted over the study period and at least five days before EEG recording and the first rTMS session. In case of severe anxiety and insomnia, zolpidem, and hydroxyzine were used.

Depressive symptoms were assessed using MADRS and CGI at baseline, week 1, and week 4. Treatment response was defined as a ≥50% reduction of MADRS total score at week four.

### EEG Apparatus and Recording

The EEG examination was carried out regularly between 8 a.m. and 10 a.m. during the baseline visit, shortly preceding the first rTMS session. We used a standard 32-channel digital EEG amplifier BrainScope (M&I, Prague) with 21 Ag/AgCl surface electrodes placed according to the international 10/20 system and cross-referenced with the electrode situated between electrodes Fz and Cz in the midline (FCz). All scalp electrode impedances were below five kΩ. The EEG recording system acquired the data with 16-bit depth, 7.63 nV/bit resolution (i.e., ∼130 bit/μV), and a dynamic range of ±250 μV. The data-sampling rate was 250 Hz and the acquired signals were filtered with digital high- and low-pass filters at 0.15 and 70 Hz, respectively. The EEG was recorded for 10 min in a sound-attenuated room with subdued lighting, with patients in a semi-recumbent position and eyes closed in a maximally alert state. During the recording, the alertness was controlled. If the patterns of drowsiness appeared in the EEG, the subjects were aroused by acoustic stimuli. The data, 10 min in duration, were collected with an on-line computer system and were stored for further computer off-line analysis.

### EEG Data Reduction and Analyses**

Prior to the data analysis, artifact detection was visually performed to exclude all epochs containing eye blink, eye-rolling artifact, head movements, muscle artifacts, and decrease in alertness or epoch in which any channel that had a voltage deflection greater than ± 100 μV. EEG reviewer was blind to the treatment outcome. In addition, remaining EEG segments were subjected to drowsiness-detection and artifact removal function using the Neuroguide software (Neuroguide^©^ NG-2.8.1, Applied Neuroscience Inc., St. Petersburg, FL), followed by the editing procedures where a template of “clean” artifact-free EEG (at least 10 sec in total duration) was selected. This template was then used to compute the matching amplitudes of EEG using flexible criteria of equal amplitudes to amplitudes that were ≤1.25 times larger. The decision as to which clean EEG sample multiplier is used was determined by the length of the sample, 60 sec as a minimum, visual inspection of the digital EEG, and when split-half reliability and test-retest reliability measurements were ≥0.95. Split-half reliability is the ratio of variance between the odd and even seconds of the time series of selected digital EEG while test re-test reliability is the ratio of variance between the first half vs. the second half of the selected EEG segments (variance=sum of the square of the deviation of each time point from the mean of the time points). Test-retest reliability >0.90 is commonly accepted in scientific literature and for a detailed description of editing, procedures see Thatcher et al. (48). Thus, from each EEG at least 60 sec (whole group mean 71.6 ± 14.9 sec); of vigilance-controlled, artifact-free, and highly reliable data were subjected to further analysis. The number of epochs, as well as the length of the samples processed, did not differ between the responders (69.1 ± 11.6 sec) and the non-responders (73.0 ± 16.7 sec) (p = 0.54). Fast Fourier transform (FFT) was used to calculate the absolute and relative power in each standard frequency bands (49). The power band analysis was based on the square rooted normalized values for the following bands: theta (4–8 Hz), alpha-1 (8–10 Hz), alpha-2 (10–12 Hz), and beta (12–20 Hz) obtained from each of the 19 electrodes (FP1/2, F3/4, F7/8, Fz, C3/4, Cz, T3/4, T5/6, P3/4, Pz, O1/2). Power asymmetry in each frequency band was calculated for inter-hemispheric (frontal F3-F4, central C3-C4, temporal T3-T4, parietal P3-P4, occipital O1-O2) and intra-hemispheric electrode pairs (on the left: frontotemporal F3-T3, F7-T3, fronto-parietal F3-P3, F7-P3; and analogously on the right: F4-T4, F8-T4, F4-P4, F8-P4). The values of asymmetry were calculated in the Neuroguide^©^ NG-2.8.1 software using the formula “power asymmetry = (A - B)/(A + B) x 200”, where A = EEG channel 1 and B = EEG channel 2 (50).

### eLORETA Calculation

Data analysis was performed using the exact low-resolution electromagnetic tomography – eLORETA (51, 52), an inverse solution technique that estimates the intracranial distribution of electrical activity (current density) in the cortex based on a three shell spherical head model co-registered with Talairach coordinates (53). We used the eLORETA-Key software (Key Institute for Brain-Mind Research, Zurich, Switzerland), available at http://www.uzh.ch/keyinst/loreta.htm. Using the eLORETA transformation matrix, cross spectra of each subject and for each frequency band were transformed to eLORETA files. This resulted in a corresponding 3D cortical distribution of the electrical neuronal generators for each subject. The computed eLORETA images reflect the cortical current density distribution in 6,239 voxels with a spatial resolution of 5 × 5 × 5 mm (52). The eLORETA algorithm has no localization of bias even in the presence of structured noise, which allows us to increase the localization accuracy, compared to the previous version of sLORETA (54). The advantage of eLORETA is that it belongs to a reference-free method of EEG analysis, therefore, determining the source distribution for EEG data is not affected by the selected electrode reference (51). LORETA is a well-proven inverse method as evidenced by its usage in numerous peer-reviewed publications (55). It has received considerable validation from studies that have combined LORETA with other more established localization methods such as the functional Magnetic Resonance Imaging (fMRI) (56–59), structural MRI (60), and Positron Emission Tomography (PET) (61–65). Furthermore, LORETA validation has been based on accepting as ground truth, the localization findings obtained from invasively implanted depth electrodes, as evident by several studies on epilepsy (66–68) and cognitive ERPs (69). Current density values were computed in eight frequency bands delta (0.5–3.5 Hz), theta (4–8 Hz), alpha-1 (8.5–10 Hz), alpha-2 (10.5–12 Hz), beta-1 (12.5–18 Hz), beta-2 (18.5–21 Hz), beta-3 (21.5–30 Hz), and gamma (35–45 Hz). These frequency bands were defined on the basis of factorial analysis of EEG records and are part of the guidelines and recommendations for the analysis of EEG records in the field of medical electrophysiological research (70).

### Statistical Analyses

The response to rTMS was defined as a ≥50% reduction of the baseline MADRS total score. Responder’s and non-responder’s demographic and clinical data were compared using the Mann-Whitney U test and Fisher exact test, as appropriate. EEG power values were transformed by square root transformation and tested using the Kolmogorov-Smirnov test. After verifying that the data was normalized, the EEG power values and asymmetries were examined using repeated-measures analysis of variance (RM-ANOVA), separately for each power band (theta, alpha-1, alpha-2, beta) with the response status as a between-subject factor (responders, non-responders) and electrode site (nineteen electrodes) or electrode pair (13 asymmetries) as a within-subject factor. Once ANOVA was significant, an unpaired t-test was applied for each electrode or electrode pair between responders and non-responders, with correction for multiple comparisons following the Benjamini-Hochberg procedure with false discovery rate q of 0.20 (71). A similar correction was applied when calculating a Pearson correlation coefficient between the pre- vs post-treatment changes in the MADRS score and normalized power values from each electrode for each frequency band.

In the eLORETA analyses, the localization of the differences in baseline activity between the group of responders and non-responders was assessed using a voxel-by-voxel unpaired t-test of the eLORETA images, based on the power of estimated electric current density. In the resulting statistical three-dimensional images, cortical voxels showing significant differences were identified using a nonparametric approach (statistical nonparametric mapping or SnPM) *via* randomizations. This randomization strategy (72) determined the critical probability threshold values for the actual observed t-values, with correction for multiple comparisons across all voxels and all frequencies. A total of 5,000 permutations were used to determine significance for each randomization test. All bands were treated simultaneously in the t-between two-sided max-statistics test guaranteeing that the family-wise type-I error did not exceed the nominal level (0.05).

## Results

### Demographic Characteristics and Clinical Measures

In our study, 9 out of 25 patients (36%) responded to the rTMS treatment. Non-responders and responders were comparable in demographic and clinical characteristics except for the baseline severity of depression (expressed by MADRS total score), which was slightly more pronounced in the non-responder group. For numerical details, see **Table 1**.

**Table 1:** Demographic and clinical characteristics of non-responders and responders to LF rTMS treatment.

	Non-responders (N = 16)	Responders (N = 9)	p-value
Age, years	47.8 (12.8)	42.0 (9.9)	0.169
Sex, F:M	13: 3	7: 2	0.835
Illness duration, months	87.1 (125.3)	83.4 (81.6)	0.301
Number of previous episodes	1.9 (2.4)	2.2 (2.4)	0.419
Duration of index episode before enrolment, weeks	40.8 (58.3)	28.7 (33.1)	0.978
Number of previous treatment trials of index episode	1.8 (1.1)	1.4 (0.7)	0.677
Baseline MADRS score	29.1 (4.2)	24.4 (2.3)	0.008
Baseline CGI score	4.4 (0.7)	4 (0)	0.136
MADRS endpoint	23.4 (5.6)	9.9 (2.7)	<0.001
MADRS change in %	-20.1 (-11.7)	-59.6 (-10.5)	<0.001
CGI score end point	3.9 (0.7)	2.0 (0.7)	<0.001
Last treatment before the enrollment	CAD-3,RIMA-1, SNRI-1, SSRI-5, TCA-1, AD+AP2-5	SSRI-4, CAD-4, AD+AP2-1	N.A.
Number of subjects taking BZD at baseline	12	5	0.20
Dose of BZD, diazepam equivalent, mg per day	14.04 (14.49)	9.49 (3.71)	0.63

Values are the mean and standard deviation (in parentheses). AD+AP2, combination of antidepressant and antipsychotic of the second generation; BZD, benzodiazepines; CAD, combination of antidepressants; CGI, Clinical Global Impression; F, females; M, males; MADRS, Montgomery and Åsberg Depression Rating Scale; N.A., not applicable; RIMA, reversible inhibitor of monoaminoxidase; SNRI, serotonin, and norepinephrine reuptake inhibitors; SSRI, serotonin reuptake inhibitors; TCA, tricyclic antidepressants.

Five out of 25 patients had experienced a first episode of MDD (responders: n = 1, non-responders: n = 4, p = 0.62, entire group: n = 25). Number of previous episodes was not statistically significant between the responders (min - max range: 0–8) and non-responders (min - max range: 0–8) (p = 0.42).

Majority of subjects (n = 16, 64%) underwent only one treatment trial prior to enrollment (no difference between groups, p = 1.0). Using a more stringent definition of resistance to treatment (≥2 previous antidepressant trials), nine subjects (36% of the sample) were considered as treatment-resistant with no differences between the groups (p = 1.0).

### Spectral Power and Asymmetry

**Figure 1:**
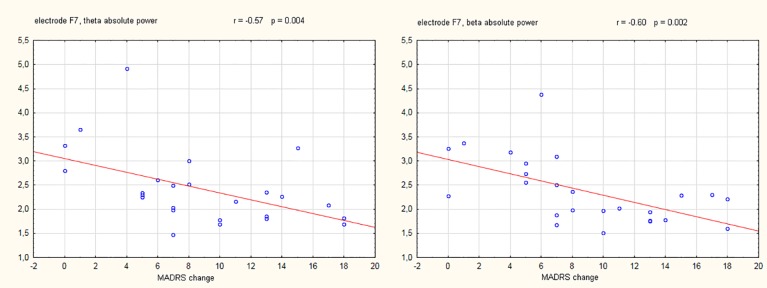
Scatterplots showing electroencephalography (EEG) absolute power values (µV2) in the theta and beta frequency band against the change of Montgomery-Åsberg depression rating scale (MADRS) score (baseline - week 4). The Pearson correlation coefficients (with corresponding p-values) for the relation between MADRS change and EEG power are also shown.

During initial data inspection, one subject from the responder's group demonstrated extremely distant (i.e., >3 standard deviations) power and asymmetry values and therefore was omitted as an outlier. Results of the repeated measures ANOVA did not reveal any significant interactions between the electrode absolute power and response status for the theta (F_(18,396)_ = 1.32, p = 0.17), alpha-1 (F_(18,396)_ = 1.21, p = 0.25), alpha-2 (F_(18,396)_ = 0.79, p = 0.71), or beta (F_(18,396)_ = 0.57, p = 0.92) frequency bands. Similarly, no significant interactions were observed for EEG asymmetries in the theta (F_(12,276)_ = 0.95, p = 0.50), alpha-1 (F_(12,276)_ = 0.73, p = 0.72), alpha-2 (F_(12,276)_ = 1,11, p = 0,35), or beta (F_(12,276)_ = 1,27, p = 0,23) frequency bands. However, a statistically significant negative correlation was observed between the change in the MADRS score (from baseline to week four) and the EEG absolute power at electrode F7 in theta (r = -0.57, p = 0.004) and beta (r = -0.60, p = 0.002) frequency bands. Scatterplots of these two significant correlations are presented in **Figure 1**.

### eLORETA

**Figure 2:**
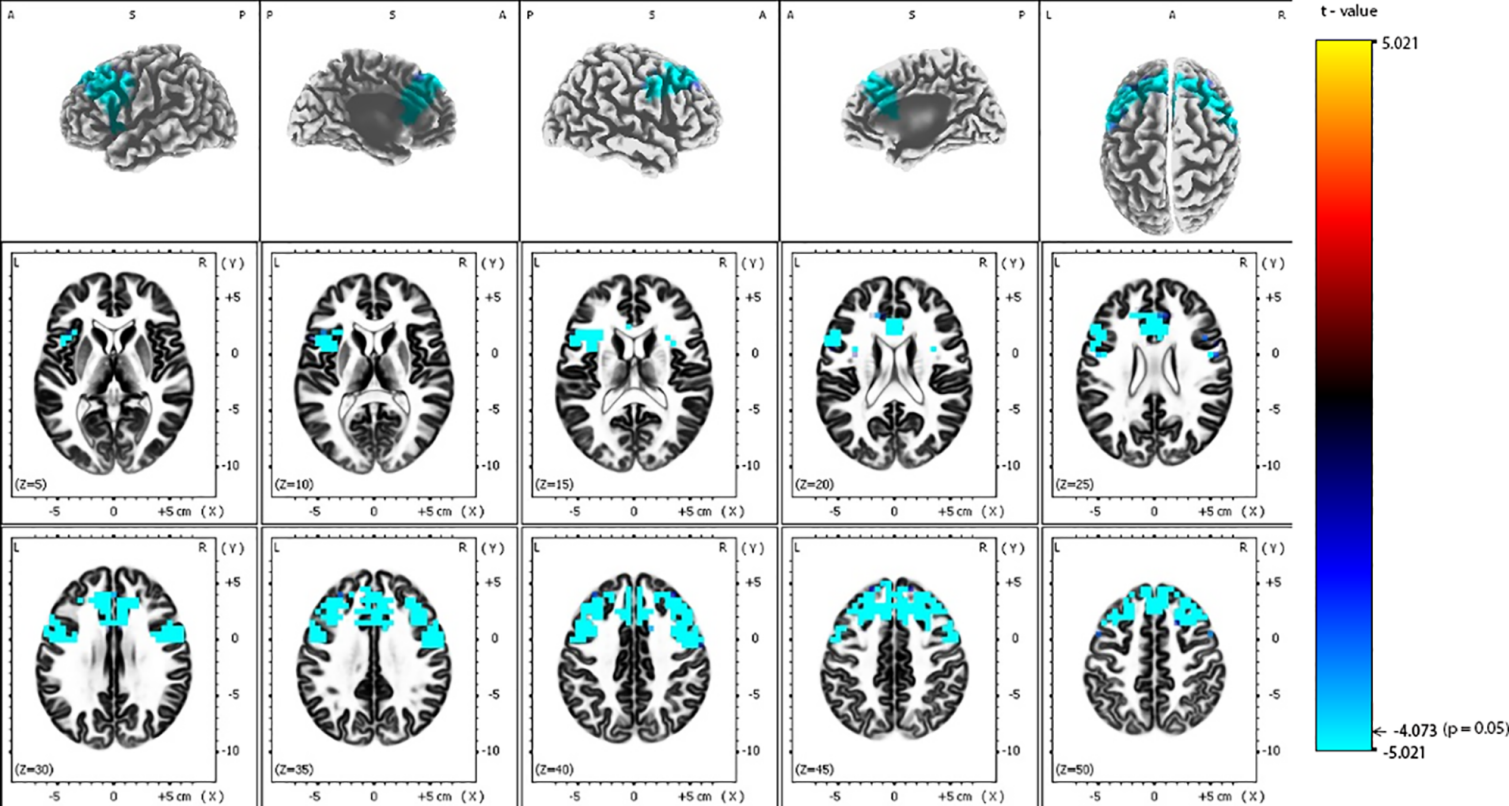
Comparison of baseline exact low-resolution brain electromagnetic tomography (eLORETA) current density for alpha-2 frequency band (10.5–12 Hz) between responders and non-responders to repetitive transcranial magnetic stimulation (rTMS) treatment. Surface-rendered (upper row) and axial sliced (middle and lower row) images depicting statistical nonparametric maps (SnPMs) are based on voxel-by-voxel independent t-tests of the eLORETA images based on the log-transformed power of the estimated electric current density. A significant decrease (p < 0.05) in the baseline alpha-2 eLORETA current density for rTMS responders is shown in the bilateral frontal lobes, insula and anterior cingulate. Structural anatomy is shown in grayscale (A, anterior; P, posterior; S, superior; L, left; R, right); x, y, z = Talairach coordinates. The threshold value (t = -4.073) for statistical significance (corresponding to p = 0.05) is reported at the color scale on the right side of the figure.

**Figure 3:**
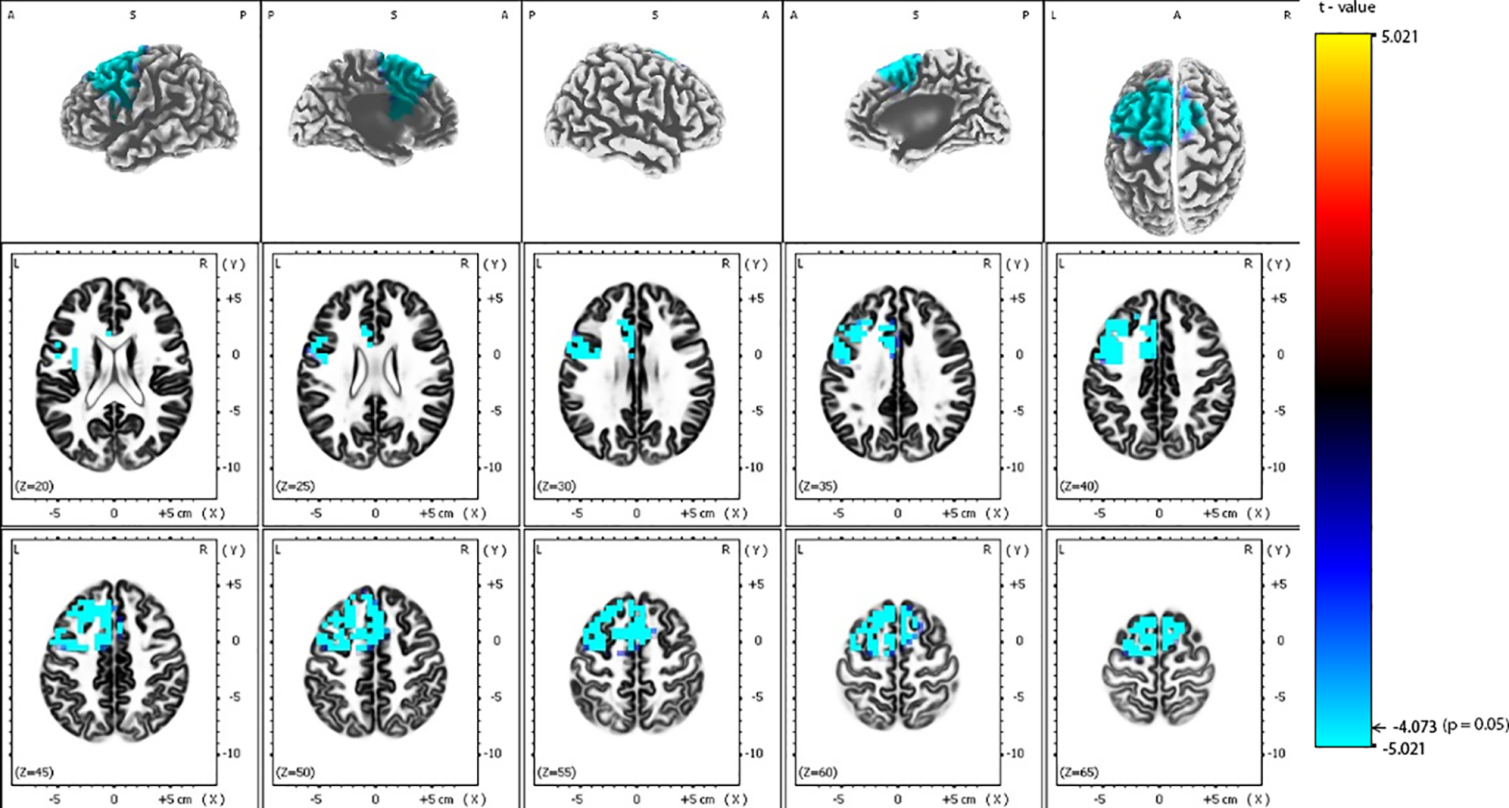
Comparison of baseline exact low-resolution brain electromagnetic tomography (eLORETA) current density for beta-1 frequency band (12.5–18 Hz) between responders and non-responders to repetitive transcranial magnetic stimulation (rTMS) treatment. Surface-rendered (upper row) and axial sliced (middle and lower row) images depicting statistical nonparametric maps (SnPMs) are based on voxel-by-voxel independent t-tests of the eLORETA images based on the log-transformed power of the estimated electric current density. A significant decrease (p < 0.05) in the baseline beta-1 eLORETA current density for rTMS responders is shown mainly in the left frontal and limbic lobes, left insula and left anterior cingulate. Structural anatomy is shown in grayscale (A, anterior; P, posterior, S, superior; L, left, R, right); x, y, z = Talairach coordinates. The threshold value (t = -4.073) for statistical significance (corresponding to p = 0.05) is reported at the color scale on the right side of the figure.

**Figure 4:**
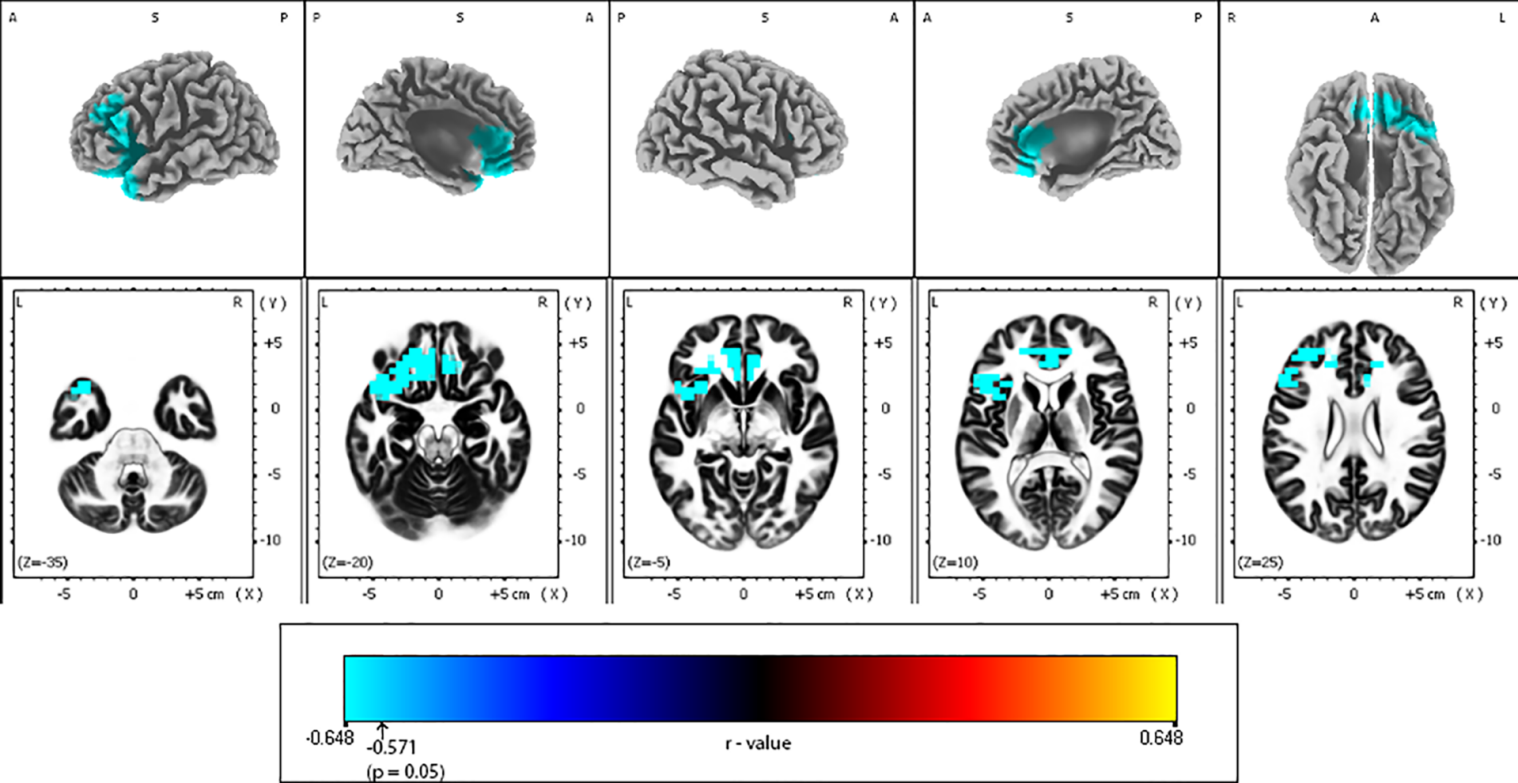
Correlations between Montgomery-Åsberg depression rating scale (MADRS) change and baseline alpha-2 exact low-resolution brain electromagnetic tomography (eLORETA) current density for the whole sample group (N = 25). Surface-rendered (upper row) and axial sliced (lower row) images depicting statistical nonparametric maps (SnPMs) are based on voxel-by-voxel regression analysis of MADRS score change (week 4 vs. baseline) on the baseline eLORETA current density in the alpha-2 frequency band (10.5–12 Hz). The significant negative effect (blue color; threshold: t = -0.571, p < 0.05) was observed between baseline alpha-2 eLORETA current density and the change of MADRS score may be seen in different brain regions: the less the alpha-2 current density mainly over left frontal and anterior cingulate regions, the better the treatment outcome. Structural anatomy is shown in grayscale (L, left; R, right; A, anterior; P, posterior; S, superior); x, y, z = Talairach coordinates. The threshold value (r = -0.571) for statistical significance (corresponding to p = 0.05) is reported at the color scale on the bottom side of the figure.

eLORETA analysis demonstrated that responders compared to non-responders, had significantly lower alpha-2 sources in the frontal lobe [Brodmann area (BA) 6, 8, 9, 32, 44, 45, 46], limbic lobe (BA 24, 33), and the insula (BA 13) (**Figure 2** and **Table 2**). Similarly, responders showed significantly lower beta-1 current densities in the frontal lobe (BA 6, 8, 9, 44), limbic lobe (BA 24), and the insula (BA 13) (**Figure 3** and **Table 3**). Most pronounced difference was found in the left middle frontal gyrus for the alpha-2 (t = -4.72, p < 0.05) and beta-1 frequency bands (t = -4.48, p < 0.05). Hemispheric distribution of the observed decreased activity in both frequency bands was localized more to the left and this asymmetry was substantially more noticeable for the beta-1 power (alpha-2: left hemisphere = 255 voxels, right hemisphere = 232 voxels; beta-1: left hemisphere = 344 voxels, right hemisphere = 53 voxels). To determine the clinical significance, we calculated the effect size based on Cohen's d for voxels with a maximum t-value for the alpha-2 (d = 1.79) and beta-1 (d = 1.74) frequency bands. To check if the abovementioned findings may reflect an unspecific improvement of depressive symptomatology or are related (and more specific) for rTMS, we calculated the correlation between the baseline MADRS and baseline eLORETA, where we did not observe any significance. However, the change in depressive symptomatology, expressed by the change in MADRS score, correlated negatively with the alpha-2 sources, mainly in the left hemisphere, with the highest negative correlation (r = -0.637; p < 0.05) localized in the anterior cingulate voxel (BA 24; x = 5, y = 30, z = 12). For more details see **Figure 4** and **Table 4**.

**Table 2:** Number of voxels, Brodmann areas and anatomical region where significant differences in baseline exact low-resolution brain electromagnetic tomography (eLORETA) current density were found for alpha-2 frequency band (10.5–12 Hz), comparing responders and non-responders to repetitive transcranial magnetic stimulation (rTMS) treatment.

All	L	R	Brodmann area	Lobe	Structure	Maximum t-statistic (x, y, z)
157	83	74	9	Frontal Lobe	Superior, Medial, Middle, Inferior Frontal Gyrus, Precentral Gyrus	-4.720 (-35, 21, 31)
148	66	82	8	Frontal Lobe	Superior, Medial and Middle Frontal Gyrus	-4.679 (35,31,44)
27	24	3	13	Insula	Insula	-4.636 (-30, 15, 13)
49	24	25	32	Frontal and Limbic Lobe	Cingulate Gyrus, Anterior Cingulate	-4.626 (10, 26, 26)
53	20	33	6	Frontal Lobe	Inferior, Medial, Middle Frontal Gyrus, Precentral Gyrus	-4.568 (15, 26, 36)
23	11	12	24	Limbic Lobe	Anterior Cingulate	-4.565 (-10, 21, 22)
14	14	0	44	Frontal Lobe	Inferior Frontal Gyrus, Precentral Gyrus	-4.498 (-50, 11, 22)
2	2	0	46	Frontal Lobe	Middle Frontal Gyrus	-4.450 (-45, 21, 22)
4	1	3	33	Limbic Lobe	Anterior Cingulate	-4.424 (0, 21, 22)
10	10	0	45	Frontal Lobe	Inferior Frontal Gyrus	-4.344 (-54, 11, 22)

Negative t-values indicate decreased activity in depressive patients responding to rTMS treatment. The number of all significant voxels (and separately for the left and right hemispheres) are given for each affected Brodmann area of brain regions. L, left; R, right; x, y, z = Talairach coordinates.

**Table 3:** Number of voxels, Brodmann areas and anatomical region where significant differences in baseline standardized low-resolution brain electromagnetic tomography (sLORETA) current density were found for beta-1 frequency band (12.5–18 Hz), comparing responders and non-responders to repetitive transcranial magnetic stimulation (rTMS) treatment.

All	L	R	Brodmann area	Lobe	Structure	Maximum t-statistic (x, y, z)
75	66	9	8	Frontal Lobe	Superior, Medial and Middle Frontal Gyrus	-4.482 (-35, 22, 50)
54	54	0	9	Frontal Lobe	Middle, Inferior Frontal Gyrus, Precentral Gyrus	-4.473 (-35, 16, 31)
191	151	40	6	Frontal Lobe	Superior, Medial, Middle Frontal Gyrus, Precentral Gyrus	-4.470 (-25, 22, 54)
14	14	0	13	Insula	Insula	-4.389 (-35, 6, 18)
36	32	4	32	Frontal and Limbic Lobe	Medial Frontal and Cingulate Gyrus, Anterior Cingulate	-4.327 (-15, 16, 31)
4	4	0	44	Frontal Lobe	Inferior Frontal Gyrus	-4.258 (-50, 11, 22)
23	23	0	24	Limbic Lobe	Cingulate Gyrus, Anterior Cingulate	-4.254 (-15, 7, 46)

Negative t-values indicate decreased activity in depressive patients responding to rTMS treatment. The number of all significant voxels (and separately for the left and right hemispheres) are given for each affected Brodmann area of brain regions. L, left; R, right; x, y, z = Talairach coordinates.

**Table 4:** Regional regression analysis of the Montgomery-Åsberg depression rating scale (MADRS) score change on the baseline alpha-2 eLORETA current density in depressive patients treated by repetitive transcranial magnetic stimulation (rTMS).

All	L	R	Brodmann area	Lobe	Structure	Maximum r-statistic (x, y, z)
22	9	13	24	Limbic Lobe	Anterior Cingulate	-0.637 (5, 30, 12)
67	34	33	32	Limbic Lobe	Anterior Cingulate	-0.634 (10, 35, 12)
27	21	6	9	Frontal Lobe	Medial, Middle and Superior Frontal Gyrus	-0.613 (-20, 35, 17)
31	31	0	13	Frontal Lobe, Insula	Inferior Frontal Gyrus, Insula	-0.611 (-30, 20, 13)
67	64	3	47	Frontal Lobe	Inferior Frontal Gyrus and Orbital Gyrus	-0.607 (-20, 29, -6)
60	46	14	11	Frontal Lobe	Inferior, Medial and Middle Frontal Gyrus, Orbital and Rectal Gyrus	-0.606 (-10, 33, -18)
24	24	0	45	Frontal Lobe	Inferior Frontal Gyrus	-0.605 (-30, 24, 3)
6	3	3	25	Frontal Lobe	Medial Frontal Gyrus	-0.601 (-10, 28, -14)
12	12	0	46	Frontal Lobe	Inferior and Middle Frontal Gyrus	-0.600 (-45, 30, 22)
17	15	2	10	Frontal Lobe	Medial and Middle Frontal Gyrus	-0.600 (-10, 39, -6)
36	36	0	38	Temporal Lobe	Superior Temporal Gyrus	-0.584 (-40, 14, -18)

Negative r-values indicate inverse correlation between the 4-week change of MADRS score and the baseline exact low-resolution brain electromagnetic tomography (eLORETA) current density values in the alpha-2 frequency band (10.5–12 Hz) for the whole sample group (N = 25). The number of all significant voxels (and separately for the left and right hemispheres) are given for each affected Brodmann area of brain regions. L, left; R, right; x, y, z = Talairach coordinates.

## Discussion

Our study findings reveal that pre-treatment decrease of alpha-2 and beta-1 sources differentiate the responders and non-responders to LF rTMS treatment, when applied to patients with major depressive disorder and who have failed to respond to at least 1 adequate antidepressant treatment in their current episode. Responders to the right-side low-frequency rTMS therapy demonstrated significantly lower current source density (CSD) in the frontal gyri, anterior cingulum, and insula, i.e., regions that have previously been related with the pathophysiology of depression (73, 74). The change in depressive psychopathology correlated negatively with alpha-2 sources in the left hemisphere, with the highest negative correlation localized in the anterior cingulate. Furthermore, none of the EEG asymmetries differentiated significantly, responders from non-responders.

Studies have confirmed the association between altered activity in the aforementioned brain regions and depressive disorder (75–77). Specifically, patients compared to healthy subjects demonstrate increased CSD in several frontal regions including anterior cingulate cortex (ACC), dorsolateral prefrontal cortex (DLPFC), medial prefrontal cortex (mPFC), and the insula (78, 79). Our eLORETA analysis reported lower activity in responders’ group in alpha-2 and beta-1 bands in similar regions. The relationship between the lower beta activity in the frontal areas in responders to the antidepressant treatment as well as a negative correlation between the lower pre-treatment alpha-2 current density in the left hemisphere with psychopathology change has been observed in previous studies. For example, Knott (15) found a smaller beta activity in the frontotemporal region that was associated with greater reductions in depressive psychopathology while an opposite relationship was observed in the frontotemporal inter-hemispheric beta coherence values. Similarly, it was observed that initially, larger values of EEG beta-1and beta-2 spectral power were associated with the high manifestation of residual depressive symptoms after treatment (29). In a recent study, Hasanzadeh et al. (30) focused on finding effective EEG features for the prediction of antidepressant rTMS treatment response. Their results refer to a specific role of beta, namely, the relative power and the sum of logarithmic amplitudes of diagonal elements of bi-spectrum in beta bands that were the most discriminating features. This corroborates with the finding that rTMS can induce a significant increase in beta-band activity in the frontal areas (80). Concerning the lower left-sided alpha-2 activity, which correlates with the changes in depressive symptomatology, we find our results comparable to ones that use a source-localization technique in order to estimate neuronal correlates of approach motivation, which is altered in depression especially when it comes to reward assessment. Pizzagalli (81) found that resting alpha-2 EEG activity in the left dorsolateral PFC regions exhibit a significant inverse correlation with reward bias, suggesting that lower alpha-2 current density is associated with lower individual reward responsiveness. Another study based on a combination of EEG and fMRI found that MDD patients with more severe depression exhibited lower left DLPFC and amygdala upper alpha EEG activity, which was corrected after antidepressant intervention (28).

Therefore, responders to the rTMS therapy, demonstrate a higher potential to increase the level of absolute power in alpha-2 and beta-1 frequency bands, which may be directly associated with therapeutic physiological response. According to previous findings, the depressive disorder could be related to lower activity in the left frontal areas (82). As the increased activity in the beta frequency band is correlated positively with the regional cerebral blood flow (83, 84), one cannot rule out that a right-sided frontal stimulation might increase the deficient metabolic activity in the left frontal lobe due to a contra-lateral adaptation process. This may manifest by increasing the absolute beta power bound to the frontal area in the case of response to rTMS treatment. As observed in an earlier study, rTMS indeed can induce a significant increase in beta activity in the frontal areas (80). However, this claim is complicated by Arrubla et al. (85), who in his recent fMRI-EEG study of resting-state demonstrated that the activity of alpha-2 and beta-1 generators in posterior cingulate correlate negatively with glutamate levels, suggesting possible increased activity also in the regions highlighted in our study. The relationship between rTMS response and lower glutamate levels was recently confirmed by fMRI spectroscopy when the responders to HF-rTMS had lower baseline concentrations of DLPFC glutamate (86). Following the abovementioned studies, the lower prefrontal and cingulate beta-1 sources found in rTMS responders in our study could be interpreted in the context of higher glutamate level in these subjects (compared to non-responders) and correspond to the reduced levels of glutamate in the ACC of severely depressed patients (87). The difference observed between the responders and non-responders in the MADRS baseline may be responsible for this discrepancy. However, since we did not find any significant correlations between baseline the MADRS and baseline eLORETA values, it is possible that our findings regarding alpha-2 and beta-1 are due to the inhibitory effect of the selected treatment protocol. Some patients with severe depression thus may be more responsive to the low-frequency protocol, which may modulate GABA/glutamate imbalance in the prefrontal and temporal cortex, while others may benefit from the high-frequency protocol, which is responsible for the direct (stimulating) increase of glutamate in these areas. In order to confirm this, it will be necessary to conduct a study analyzing resting-state alpha-2 and beta-1 sources using high-resolution EEG and MR spectroscopy measurement before, during, and at the end of LF-rTMS treatment.

However, this does not explain whether the detected EEG and eLORETA profile reflects the more general index of a response outcome. Theoretically each of the source bands (alpha-2 and beta-1), in relation to certain brain areas, does not differ in their relationship to the specificity of the physiological changes that LF-rTMS can induce. This would be in line with our observations, where psychopathology change correlated with alpha-2 but not beta-1, suggesting that alpha-2 and beta-1 source generators may differ in their sensitivity to rTMS intervention. However, this was not confirmed by the above-mentioned findings where changes in both the bands occurred in different treatment interventions. Further studies with an in-depth analysis of the neuronal sources of these frequency bands in responders, non-responders, and healthy volunteers based on e.g. simultaneous EEG-fMRI assessment, and taking into account different stages of anti-depressive treatment, may provide a more comprehensive answer to this question.

## Limitations

The response rate in our study is relatively low. However, Berlim´s and Cao´s meta-analyses (6, 88) showed similar response rates as observed in our study. We cannot exclude the higher rate of positive results using increased motor threshold (110%–120%) and a number of stimulations (extended course to 30 stimulation) recommended in the recent guidelines (89). There are also modified or new coil positioning techniques such as 5.5 or 6 cm anterior to the motor cortex (e.g., the centimeter rule), coil placement on F3 position according to the International 10–20 system, use of stereotactic frames, and neuroimage-guided frameless positioning technologies (90) which can increase the response rate. The results of this study thus should be interpreted with caution due to its limited sample size (only 9 subjects in the responder’s group). It should be noted, however, that we experienced difficulties during the recruitment process due to the enrolment restrictions, especially the termination of medication being administered and the approval of the rTMS course application. Future studies conducted in a larger clinical sample may further support our findings. Furthermore, we did not include a placebo arm (sham stimulation without any medication) as participation of patients with MDD without any medical treatment is ethically unacceptable and not approved by the Prague Psychiatric Center Institutional Review Board, especially for treatment of patients who failed to respond to at least one adequate antidepressant treatment in their current episode. Another limitation of the EEG part of the study is the use of a relatively small number of electrodes, however, EEG assessments in a real clinical setting are usually conducted with 19 or 21 scalp electrodes. Although the number of EEG electrodes is related to the precision of source estimation, several studies indicate that a reliable LORETA/eLORETA estimation can be achieved with just 19 channels (91–96). Nevertheless, eLORETA accuracy still depends to an extent on the EEG montage density and the relatively small number of electrodes that we were limited to suggest some caution in interpreting our findings. Higher density EEG caps (e.g., at least 64 electrodes) can provide a more accurate source localization. Finally, the neuropsychological assessment had not been conducted in the patients prior to rTMS therapy. Future research conducted in a larger sample adopting initial neuropsychological testing might clarify the association between the potential benefit of rTMS therapy and the specific neuropsychological profile of depressive patients.

## Conclusion

Our study revealed that the patients with major depressive disorder, responding to the right-side LF rTMS therapy, differed significantly in alpha-2 and beta-1 current density sources, which were decreased in the frontal gyri and limbic structures (anterior cingulum and insula) when compared to the non-responders. Scalp-derived QEEG measures revealed a negative correlation between the beta absolute power at the left frontal electrode F7 and the change in depressive symptomatology. However, none of the EEG asymmetries differentiated significantly the responders from non-responders. Future studies may validate our results in larger and more diverse samples, including cognitive and genetic testing related to depressive disorder.

## Data Availability Statement

The datasets generated for this study are available on request to the corresponding author.

## Ethics Statement

The study involving human participants was reviewed and approved by the ethical committee of the Prague Psychiatric Centre/National Institute of Mental Health, Czech Republic. The patients/participants provided their written informed consent to participate in this study.

## Author Contributions

PV analyzed EEG data, managed literature searches, discussed results, and drafted the manuscript. MBa designed the study, participated in the clinical part of the project, and revised the manuscript. TN designed the study, participated in the clinical part of the project, undertook statistical analyses, and revised the manuscript. MBr designed the EEG part of the study, coordinated project activities, analyzed data, discussed results, and drafted and revised the manuscript.

## Funding

The study was supported by grants from the Czech Ministry of Health, no. AZV 15-29900A and 16-31380A; by the projects PROGRES Q35 and 260388/SVV/2019 of the Third Faculty of Medicine, Charles University and by the project ““Sustainability for the National Institute of Mental Health” (NPU4NUDZ) under the grant number LO1611 with a financial support from the Ministry of Education, Youth and Sports of the Czech Republic under the NPU I program.

## Conflict of Interest

The authors declare that the research was conducted in the absence of any commercial or financial relationships that could be construed as a potential conflict of interest.
